# Integrity Assessment of a Hybrid DBS Probe that Enables Neurotransmitter Detection Simultaneously to Electrical Stimulation and Recording

**DOI:** 10.3390/mi9100510

**Published:** 2018-10-10

**Authors:** Danesh Ashouri Vajari, Maria Vomero, Johannes B. Erhardt, Ali Sadr, Juan S. Ordonez, Volker A. Coenen, Thomas Stieglitz

**Affiliations:** 1 Laboratory for Biomedical Microtechnology, Department of Microsystems Engineering (IMTEK), University of Freiburg, Georges-Kohler-Allee 102, 79110 Freiburg, Germany; maria.vomero@imtek.uni-freiburg.de (M.V.); johannes.erhardt@imtek.uni-freiburg.de (J.B.E.); ali.sadr.63@gmail.com (A.S.); jsordonez@posteo.net (J.S.O.); thomas.stieglitz@imtek.uni-freiburg.de (T.S.); 2 BrainLinks-BrainTools Cluster of Excellence, University of Freiburg, Georges-Kohler-Allee 79, 79110 Freiburg, Germany; volker.coenen@uniklinik-freiburg.de; 3 Indigo Diabetes N.V., Bollebergen 2B box 5, B-9052 Gent, Belgium; 4 Bernstein Center Freiburg, University of Freiburg, Hansastrasse 9a, 79104 Freiburg, Germany; 5 Department of Stereotactic and Functional Neurosurgery, University Medical Center Freiburg, Breisacher Strasse 64, 79106 Freiburg, Germany; 6 Faculty of Medicine, University of Freiburg, 79110 Freiburg, Germany

**Keywords:** deep brain stimulation, fast scan cyclic voltammetry, dopamine, glassy carbon electrode, magnetic resonance imaging

## Abstract

Deep brain stimulation (DBS) is a successful medical therapy for many treatment resistant neuropsychiatric disorders such as movement disorders; e.g., Parkinson’s disease, Tremor, and dystonia. Moreover, DBS is becoming more and more appealing for a rapidly growing number of patients with other neuropsychiatric diseases such as depression and obsessive compulsive disorder. In spite of the promising outcomes, the current clinical hardware used in DBS does not match the technological standards of other medical applications and as a result could possibly lead to side effects such as high energy consumption and others. By implementing more advanced DBS devices, in fact, many of these limitations could be overcome. For example, a higher channels count and smaller electrode sites could allow more focal and tailored stimulation. In addition, new materials, like carbon for example, could be incorporated into the probes to enable adaptive stimulation protocols by biosensing neurotransmitters in the brain. Updating the current clinical DBS technology adequately requires combining the most recent technological advances in the field of neural engineering. Here, a novel hybrid multimodal DBS probe with glassy carbon microelectrodes on a polyimide thin-film device assembled on a silicon rubber tubing is introduced. The glassy carbon interface enables neurotransmitter detection using fast scan cyclic voltammetry and electrophysiological recordings while simultaneously performing electrical stimulation. Additionally, the presented DBS technology shows no imaging artefacts in magnetic resonance imaging. Thus, we present a promising new tool that might lead to a better fundamental understanding of the underlying mechanism of DBS while simultaneously paving our way towards better treatments.

## 1. Introduction

**Figure 1:**
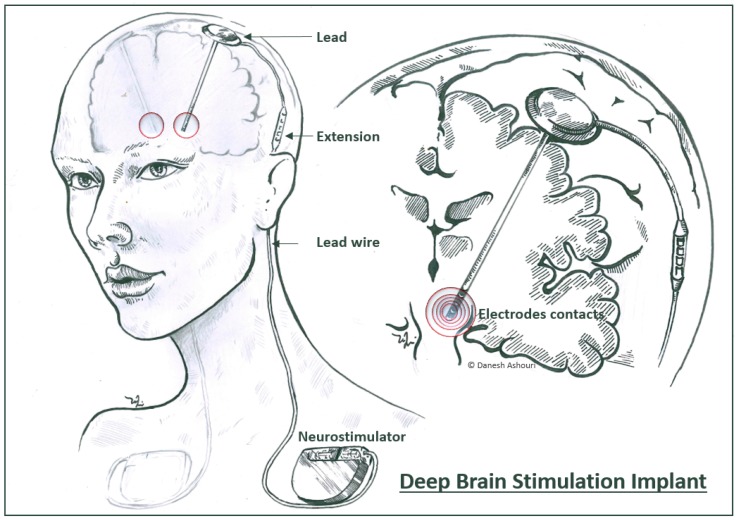
Illustration of a deep brain stimulation device implanted in a patient. A deep brain stimulation implant consists of four main components; electrode contacts, lead, lead wire, extension part and the implantable pulse generator (IPG or neurostimulator). A conventional DBS probe is featured with four annular active sites/contacts to deliver electrical current to the target tissue (diagram by D. Ashouri Vajari).

Deep brain stimulation (DBS) is a widely used treatment for neurologic disorders such as Parkinson’s disease, tremor, dystonia, and epilepsy [1,2,3]. Promising research is performed in other psychiatric disorders like depression and others [2,3,4,5]. In principle, DBS mainly activates nerve cells in certain brain regions (specified by the application and thus the anatomical placement) by delivering an electrical stimulus through conductive sites (Figure 1) [5]. Precision in stimulating the target area—and therefore having a defined volume of activated tissue—plays an essential role in the success of the treatment. However, the current technology used for DBS is relatively outdated and comes down to some limitations. The rather large size of the contact sites (annular electrode contacts; 1.27 mm diameter, 1.5 mm height; i.e., 6.0 mm^2^ surface area) and hence, the large volume of activated tissue in the case of conventional DBS probes, can cause the flow of current to reach outside of the target regions and also sometimes high stimulus intensities and/or misplaced leads can increase the chance of inadvertently stimulating the functional environment and hence, result in unwanted stimulation-related side effects [6,7]. 

One approach to improve precision in stimulation is by decreasing the dimension of the stimulating electrodes accompanied by a higher channel count, in order to minimize the desired tissue volume to be excited [5,8,9,10]. Recently some efforts have been made to increase the channel density of DBS probes (Table 1) associated with spatial selectivity; e.g., Toader et al. (Sapiens probe)—to minimize side effects by optimizing the specificity and reducing the volumes of activated tissue [11,12]. A higher channel density enables the activation of single miniaturized sites (individually or in combination with other contacts) and can provide a higher degree of freedom by facilitating asymmetrical stimulation and steering the stimulation field [12,13]. 

While the accuracy of DBS outcome is supported by imaging [14], modeling [15], navigation [16,17], and microelectrode recording [18], feedback of the awake patient during surgery is requested [19] in some implantation paradigms. Even though no statistical significant differences have been reported in clinical output between electrode placement in awake and anaesthetized patients, this topic is discussed highly controversially [20,21,22]. In addition to verbal feedback, which lacks accurate informative features of the undergoing biological events, acquiring more quantitative information, for example, monitoring the neurotransmitter levels can be beneficial to further advance our understanding of the applied methodology. Fast scan cyclic voltammetry (FSCV) is an electrochemical method that provides information about the relative changes of the electroactive neurochemicals in a sub-second range, both in vitro and in vivo [23]. The most commonly used electrodes for this measurement are carbon fibers microelectrodes (CFMs) because they provide high sensitivity and selectivity in detection of neurotransmitters [24]. However, CFMs are brittle and applicable for intrasurgical application only and therefore [25,26], cannot be incorporated into current DBS probes. 

Precise implantation is another essential aspect of a successful treatment that is assisted by utilizing a stereotactic frame and imaging techniques. Magnetic resonance imaging (MRI) is considered as the gold standard for post implantation electrode placement verification [27]. However, the magnetic susceptibility artefacts of the conventional DBS probes can impede a precise localization [28]. Besides, there is great interest in the combination of DBS and functional MRI (fMRI) to acquire more data and a deeper understanding of the brain [29,30]. fMRI is more sensitive to susceptibility artefacts than standard MRI, hence the diagnostic value in the vicinity of conventional DBS probes is limited due to signal voids [31]. 

In recent years many efforts have been made to address multiple limitations in (a) the development of reliable small devices and (b) having access to biomarkers such as neurotransmitter activities. However, yet in the field of neuromodulation, there is a lack of probes that combine such multi-modalities to enable a comprehensive coverage of the known biological informative features (e.g., neural activity and neurotransmitter levels) in order to further investigate the underlying mechanisms of brain disorders. Aligned with these challenging aspects, we have previously presented the application of both polyimide thin-film devices [32,33,34,35,36,37] as well as silicone rubber based electrodes in the field of neural prosthesis. Furthermore, the usage of carbon-based material (e.g., glassy carbon [38,39,40] and laser induced carbon [41,42]) as a multimodal interface that enables electrical stimulation, monitoring neural activity in addition to neurochemical detection, was also introduced. The presented technologies on sight can potentially give new directions in various fields of brain research. However, yet there has not been a device which combines these advances reliably and in compliance with the conventional technologies. The presented work focuses on deep brain stimulation (DBS) as a widely used clinical technique and aims to further advance its capabilities by addressing the main associated limitations. 

In this study we present a design of a hybrid DBS probe, a combination of a polyimide based thin-film devices and a silicone rubber substrate, which in addition to the basic modalities (i.e., electrical stimulation and local field potentials (LFP) recording) offers the possibility of performing FSCV to monitor the relative changes of the in situ neurotransmitter levels. The fabrication of the probe was realized by utilizing the previously published Carbon-Microelectromechanical systems (C-MEMS) technology [39,40] that allows the incorporation of glassy carbon (GC) electrodes onto a PI substrate [33,34,35,43] and combining the fabricated thin-film device with the flexible silicone rubber tubing. 

## 2. Materials and Methods 

### 2.1. Electrode Fabrication

#### 2.1.1. C-MEMS Technology and Fabrication of Glassy Carbon

**Figure 2:**
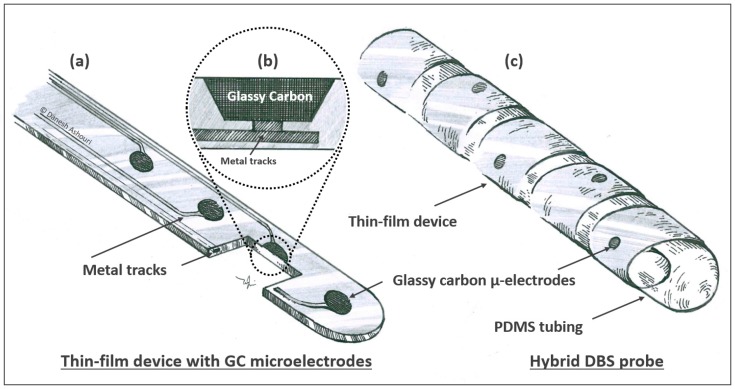
Schematic of the hybrid deep brain stimulation (DBS) probe. (**a**) Thin-film device with glassy carbon (GC) microelectrodes (50 um in diameter) embedded into a polyimide substrate. The active sites are distributed homogeneously along the length of the foil; (**b**) Cross-sectional view of the interface between the glassy carbon and the metal tracks (**c**) Assembled hybrid probe showing the spiral design of the wrapped thin-film device shown in (**a**) around the silicone-rubber tubing (diagram by D. Ashouri Vajari).

The thin-film device with GC electrodes was fabricated using a method previously described elsewhere [38,39,40]. In short, the high aspect ratio photoresist SU-8 (MicroChem, Westborough, MA, USA) was used as a precursor for 50 um-diameter glassy carbon electrodes, which was resulted from pyrolyzation in a nitrogen atmosphere at 1000 °C. Then a layer of polyimide (PI, U-Varnish-S, UBE Industries, Ltd., Ube, Japan) was spun onto the carrier wafers and subsequently, the PI layer was etched above these electrodes to provide access. Metallization of the conducting tracks and a second layer of PI for insulation followed. This process yielded 8 µm-thick devices (Figure 2). Zero insertion force (ZIF) connector was the used interconnection technology, which facilitates a simple and quick usage for on-bench measurements.

#### 2.1.2. Assembly of the Hybrid Probe

The assembly of the hybrid probe consisted of two steps: first, the thin-film device was released from the wafer and rinsed in isopropanol and DI water, together with a silicone tubing featuring an outer diameter of 1.19 mm. Then the thin film device tip was fixed to the open end of the silicone tubing using silicone rubber adhesive (silicone rubber, DC 3140, RTV coating, Dow Corning, Midland, MI, USA) which was then left to cure for six hours. A tungsten rod was used as a stylet to keep the tubing straight. A thin layer of silicone rubber adhesive was applied onto the surface of the tubing to ease the assembly of the probe. The thin-film device was held at a 45° angle with respect to the tubing and carefully wrapped around (by turning the tubing clockwise around its longitudinal axis). The assembled probes were then left to cure at room temperature for 24 h. 

### 2.2. Electrochemical Characterization

The electrochemical performance of all the fabricated electrodes was evaluated by means of cyclic voltammetry (CV) and electrochemical impedance spectroscopy (EIS). For both measurements, a three electrode configuration was used where a silver|silver-chloride electrode and a standard Pt electrode were utilized as the reference and counter electrode, respectively [44]. For the CV measurements, a conventional triangular waveform was used in which the vertex potential was swept in between −0.9 V to 1.1 V at a scan rate of 50 mV/s. Prior to each CV characterization, a cleaning step (6 cycles using the same parameters at a scan rate of 250 mV/s) was introduced. Following the CV, in order to study the impedance behavior of the electrodes under test, all the samples were subjected to EIS measurements. To perform the EIS measurements, a sinusoidal excitation of 10 mV_pp_ between 1 Hz and 100 kHz was applied. Both methods were realized by utilizing a potentiostat in combination with a frequency analyzer (Solartron 1260–1287 by Solartron Analytical, Farnborough, Hampshire, UK). After each measurement, the working electrode was rinsed in DI water.

### 2.3. Electrical Stimulation 

Electrical stimulation was used to study the performance of the hybrid DBS probe when subjected to the clinically relevant stimulation paradigms. The used stimulation parameters were adapted from a used set for treating Parkinson’s disease. A charge balanced, rectangular, cathodic first stimulation waveform at 130 Hz repetition rate was used to conduct the electrical stimulation. This experiment was performed using a Plexon stimulator (Neurotechnology Research Systems) and an in-built circuit to subtract the voltage drop over the access resistance. The needed charge density for the hybrid probe (electrode size: 50 μm in diameter) was 7.2 μC/cm^2^ charge density/phase (adapted from the Parkinson’s treatment). Phosphate buffered saline (PBS with pH = 7.4) was used as the carrier electrolyte. For this experiment, a two electrode configuration was used in which a large area (~1 cm²) stainless steel electrode served as the counter electrode. 

### 2.4. Neurochemical Measurements

**Figure 3:**
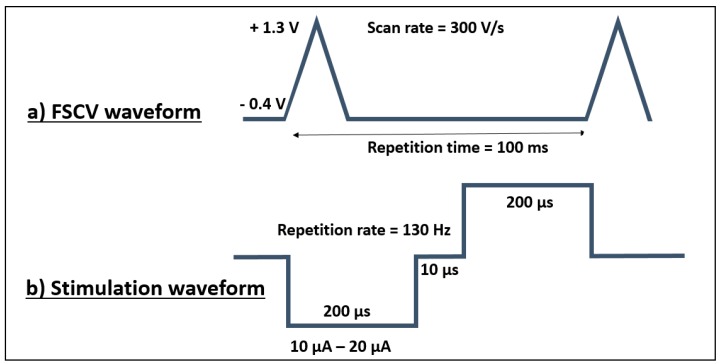
Schematic of the used waveform for electrical stimulation and for fast scan cyclic voltammetry; (**a**) Fast scan cyclic voltammetry (FSCV) waveform, the 100 ms delay with the −0.4 V holding potential increases the possibility of accumulation of the dopamine molecules prior each scan, the temporal resolution of the detection system is limited by the delay time between two scans; (**b**) biphasic, charged balanced, cathodic first waveform used for the pulse test.

Fast scan cyclic voltammetry is an electrochemical method which facilitates high-resolution real-time analyte measurements; e.g., dopamine [45,46]. FSCV was realized using a potentiostat (Invilog systems Ltd., Kuopio, Finland) providing a triangular waveform in which the vertex potential was swept between −0.4 V and 1.3 V at 10 Hz repetition rate with a scan rate of 300 V/s (Figure 3). The two electrode configuration was used to form the electrochemical cell where a chlorinated silver wire served as the reference electrode [46]. In FSCV, the applied triangular waveform at the sensing electrode causes the electroactive compounds in the vicinity of the electrode surface to undergo oxidation/reduction [47]. The applied potential results in oxidizing the dopamine molecule to dopamine-o-quinone by delivering two electrons which is then followed by the reduction of the remaining dopamine-o-quinone back to dopamine by sweeping the voltage in the opposite direction [48]. The occurred oxidation-reduction (redox) reactions generate a current that is linearly proportional to the amount of electroactive compounds in the vicinity of the surface of the sensing electrode. Assuming the sensing electrode stays consistent in its properties, the potential ranges in which the generated redox current appear to differ depending on the neurochemical of interest [49,50]. Therefore, using the magnitude of the generated oxidation peak and its potential range, an estimation on the nature of the present electroactive compounds and their concentration can be obtained. The temporal resolution of this detection system is limited by the applied delay time in between two FSCV scans (in the order of 100 ms) which covers a big range of both the low frequency (1–5 Hz) tonic activity and the high frequency (≥ 20 Hz) phasic activity modes of the dopaminergic neurons [51,52]. In this study, glassy carbon was the material of choice used as the sensing interface.

The collected data were digitally subtracted to eliminate the impact of the capacitive component of the redox reactions. All the data processing needed for this experiment was done using MATLAB (MATLAB and Statistics Toolbox Release 2012b, The MathWorks, Inc., Natick, MA, USA). Prior to and after each experiment, all the under study active sites were electrochemically characterized (see Section 2.2). After the initial characterization of electrodes, all the testing sites were cycled for 20 min using the given setup (Figure 3.). This baseline measurement was essential to improve the signal quality by decreasing the fluctuations of the background current (µA range) and therefore, to bring more stability and visibility to the faradic components of the signal (in some tens of nA range). The calibration was then realized by applying the known concentration of the prepared dopamine stock solution to the under-test electrochemical cell. The applied concentrations were ranged in a manner that would cover both the fundamental and the application targeted purposes. Six different concentrations were used to perform the calibration: 100 nM, 500 nM, 1 µM, 2 µM, 3 µM, and 5 µM. After each registration of dopamine, a 40 s delay was introduced before proceeding with the next injection. After each calibration, the tested sites were initially rinsed with DI water and cycled in PBS using given waveforms in order to ensure having no residues of the solution left on the surface of the electrode. The cleaned electrodes were then taken to the next characterization step by means of EIS and CV. 

### 2.5. Magnetic Resonance Imaging

A hybrid probe and a 3389 DBS lead (Medtronic^®^, Minneapolis, MN, USA) were cast in a 1% agarose phantom, mimicking the MRI contrast of grey matter [53,54]. The sample was then placed in a receive only head coil of a 1.5 T MRI system (MAGNETOM Tim Symphony, Siemens Healthcare GmbH, Erlangen, Germany) where the leads were oriented along the axis of the bore while coiling excess length of the DBS lead in the transversal plane similar to the arrangement recommended by the manufacturer. The samples were then imaged employing the following three standard imaging sequences: Turbo Spin Echo (spatial resolution 0.7 × 0.7 × 2 mm^3^; repetition time 2000 ms; echo time 9.3 ms; 4 averages), Inversion Recovery (spatial resolution 0.7 × 0.7 × 3 mm^3^; repetition time 4000 ms; echo time 74 ms; 4 averages) and Gradient Echo (coronal and transversal: spatial resolution 0.75 × 0.75 × 2 mm^3^; repetition time 10 ms; echo time 5.1 ms; 32 averages). Additionally, images were taken using Echo Planar Imaging (spatial resolution 0.9 × 0.9 × 3.2 mm^3^; repetition time 145 ms; echo time 15 ms; 32 averages) to illustrate the imaging artefacts that would have to be taken into account during fMRI.

## 3. Results

### 3.1. Fabrication and Assembly of the Hybrid Probe 

**Figure 4:**
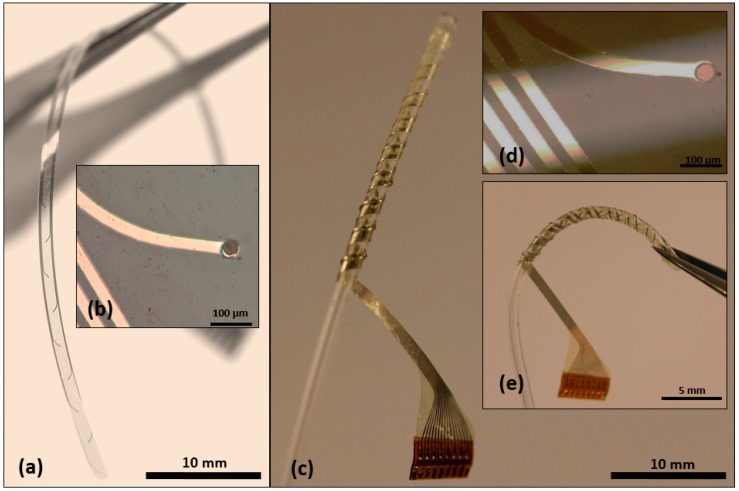
Representation of the fabricated thin-film device and the assembled hybrid probe; (**a**) cleanroom fabricated GC thin-film electrode featuring 16 active channels and zero insertion force (ZIF) interconnection in hybrid assembly; (**b**) a close-up of the thin-film device and the GC sites:, the dark disk-shaped site represent a glassy carbon interface present at the end of the metal tracks; (**c**) the assembled hybrid DBS probe; (**d**) a representative image of the electrode surface after wrapping the thin-film device around the silicone rubber tubing—no deformation/delamination on the glassy carbon interface was observed; (**e**) the hybrid assembly offering a higher stability to the thin-film device by not only introducing more flexibility and also by allowing for stretch without damage.

Fabrication and assembly of the hybrid (DBS) probes were successful (Figure 4). The thin-film devices with GC microelectrodes smoothly followed the curvature of the silicone tubing and adhered to it. Silicone rubber was applied around the tubing before wrapping the thin-film component, and to fix the thin-films in place and fill the eventual voids between the polyimide and the tubing. No sharp edges remained. The final result was thus a uniform and solid composite device with GC electrodes and PI on the external ‘shell’ and soft silicone rubber in the core. 

### 3.2. Electrochemical Characterization

**Figure 5:**
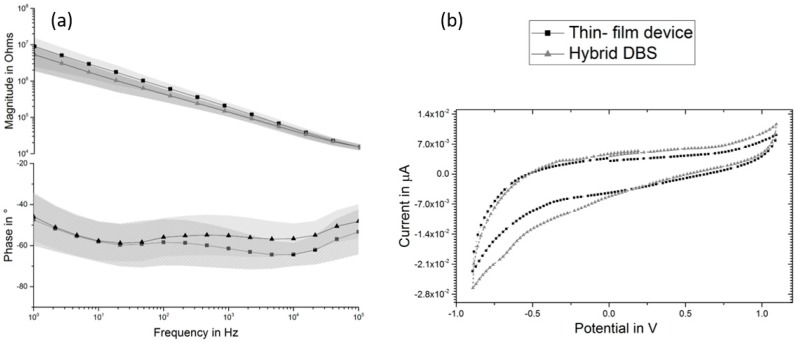
Electrochemical characterization of the fabricated glassy carbon electrodes prior to and after the wrapping: (**a**) The conducted electrochemical impedance spectroscopy (EIS) showing the influence of the wrapping on the performance of the electrodes in comparison to the un-wrapped electrode; (**b**) The representative CV diagram presenting the resulted characterizations of the thin-film device and hybrid probe.

The performed EIS measurements on the thin-film devices and the hybrid probes revealed an impedance value of 67 kΩ (@1 kHz) and 13.9 kΩ (@1 kHz), respectively. The presented data were obtained by calculating the average values and the standard deviation of the characterized electrodes (n = 10). The averaged phase value was found to be −64° (@1 kHz) and −56° (@1 kHz), respectively (Figure 5a). CVs of both, thin-film device and the hybrid probe (Figure 5b), exhibit comparable shape with no oxygen/hydrogen evolution at the two edges of the vertex potentials (i.e., −0.9 and 1.1 V (Figure 5b)) but increased in area under the curve, indicating more redox reactions during cycling and more capacitive behavior during EIS at frequencies above 100 Hz. 

### 3.3. Influence of Electrical Stimulation 

The performed electrical stimulation (the stimulation parameters are given in Figure 3b) resulted in a voltage across the phase boundary with a magnitude of 500.6 mV (averaged for n = 10). To further evaluate the performance of the hybrid probe, active sites were subjected to 81 μC/cm^2^ charge density per phase, which resulted in a phase boundary potential of 1.9 V. 

### 3.4. Neurochemical Measurements

**Figure 6:**
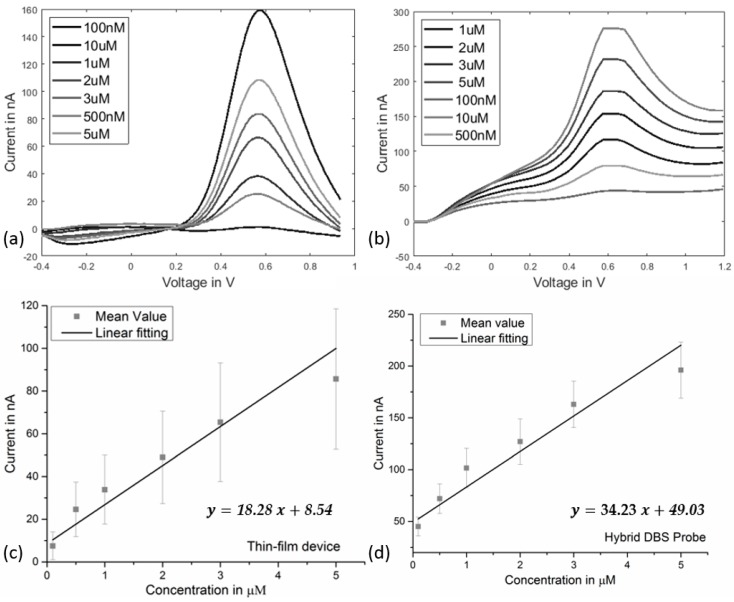
In vitro calibration curves of the conducted FSCV experiments for the thin-film devices as well as the hybrid probes; (**a**) representative FSCV diagram using thin-film device; (**b**) representative FSCV diagram of one of the calibrated hybrid probes with the calculated standard deviations; (**c**) linear fitting of the calibration curve for the thin-film device (n = 3); (**d**) linear fitting of the calibration values based on the calculated average for the hybrid assembly (with the calculated standard deviations ; n = 6).

All the samples underwent the same baseline measurements in the FSCV experiment in which electrodes were cycled for 20 min using the given waveform (Figure 3) in order to obtain a stable background current. After reaching a relatively stable current value over time, different dopamine concentrations were applied and by averaging the generated oxidation peak, the calibration values for single electrode were calculated. The overall sensitivity was then obtained by applying the linear regression over the calculated average values including the standard deviation values of the grouped samples prior to and after wrapping. The calibration of the device prior to wrapping revealed a sensitivity of 18.28 nA/µM (n = 3 probes). The calibration of the individual single electrodes, however, delivered slight deviations in the sensitivity value (Figure 6, standard deviation). The calibration of the hybrid probe (after wrapping) resulted in a sensitivity of 34.23 nA/µM. Regardless of the observed differences in the absolute sensitivity values, all samples were found sensitive to the changes of applied concentration of dopamine.

### 3.5. Magnetic Resonance Imaging of a DBS Probe and the Hybrid Probe

**Figure 7:**
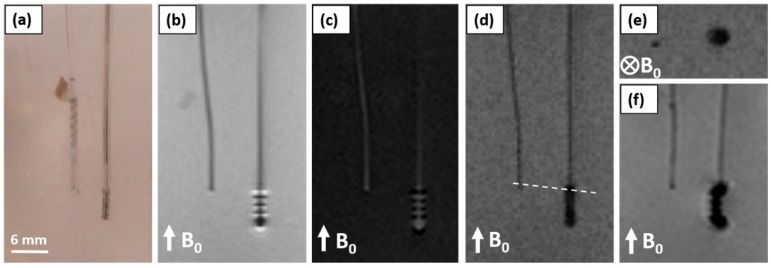
Comparison of the hybrid probe with a conventional DBS probe with respect to implant localization and imaging artefacts due to the electrode material in common MRI sequences using a 1.5 T scanner; (**a**) Photograph of the hybrid probe (left) and the conventional 3389 Medtronic^®^ DBS probe (right) in a 1% agarose phantom; (**b**) Coronal view using a Turbo Spin Echo sequence; (**c**) Coronal view using an Inversion Recovery sequence; (**d**) Coronal view using a Gradient Echo sequence; (**e**) Transversal view along dashed line in (**d**) using a Gradient Echo sequence; (**f**) Coronal view using an Echo Planar Imaging sequence. Both devices have a similar diameter range (hybrid probe 1.19 mm; conventional DBS 1.27 mm) the displayed diameter at the tip of the conventional DBS probe, however, appears larger due to susceptibility artefacts.

The performed MRI showed the hybrid probe in all four employed MRI sequences clearly visible without any image distortions or signal losses due to its metal components. By contrast, the conventional DBS probe showed susceptibility artefacts around the electrode active sites where the image is distorted and signal voids occur (Figure 7). In the coronal acquired images (b–d), the lateral dimension of the commercial DBS lead tip appears three times as wide as its dimensions. The Echo Planar Imaging sequence (Figure 7f) showed twofold larger artefacts for the conventional DBS lead as compared to the previous sequences. The cross-section in Figure 7e shows the conventional DBS lead tip as an area fourfold of its dimensions.

## 4. Discussion

In this manuscript, we have combined technologies to design and manufacture a prototype of a multimodal DBS probe. Used technologies are all state of the art in microsystems engineering. Therefore, the reduction of electrode size and increase in channel count goes hand in hand with the opportunity to transfer this study into a medical device. This would increase manufacturing readiness level together with the technology readiness level in preclinical studies and clinical trials with few iterations, only. We were able to manufacture a proof-of-concept prototype and to characterize the hybrid probe in vitro. Since glassy carbon is a relatively stiff and brittle material [55], special attention has been laid on the influence of wrapping on the thin-film electrode integrity and its performance during electrochemical characterization. 

### 4.1. Fabrication and Assembly of the Probe

Cleanroom fabrication steps were successfully carried out and the final devices met the expectations in terms of optical appearance and electrochemical properties. The suggested design in this work targets an exemplary probe architecture. Nevertheless, having the advantage of cleanroom fabrication, the hybrid probe can be tailored for different applications. Furthermore, glassy carbon as the interface of the probe enables a multimodality of every active site to conduct electrical stimulation, record from neural activity, and also monitor neurotransmitters like dopamine. For this proof-of-concept probe, a manual fabrication technique was used, however, the development of an automated production would be an essential step for increasing the manufacturing readiness level of the concept on its way to a medical device. 

### 4.2. Fast Scan Cyclic Voltammetry

According to our FSCV experiments, both sample types, i.e., the unwrapped and wrapped thin-film devices, showed sensitivity towards the applied concentration of dopamine in the calibration experiment. The sensitivity value was calculated to be 18.28 nA/µM and 34.23 nA/µM for the thin-film device and the hybrid probe, respectively. Among all the calibrated samples, the sensitivity trend towards the applied concentration was clear. However, the magnitude of the generated oxidation peaks varied slightly in between different electrodes. Nevertheless, individual sensing sites showed consistent performance in sensing dopamine considering their peak potential and the magnitude of oxidation current. Differences in performance among the carbon electrodes can be due to different surface porosity achieved during pyrolysis. The samples, in fact, were not polished or treated (i.e., activated) in any way before conducting the dopamine detection experiments and thus differences in surface morphology and surface oxidation are expected [38,39]. Nevertheless, the overall performance of the hybrid probe in the given configuration was found promising and opens new directions in neurochemical monitoring during neurosurgical interventions and treatments using DBS.

### 4.3. Electrical Stimulation

The performed electrical stimulation showed that the hybrid probes are capable of delivering a similar charge as the conventional DBS probes without exceeding their water window. The result of voltage transient in response to the applied waveform was found in between the water window of glassy carbon interface and even by increasing the delivered charge by a factor of ~11, the potential across the phase boundary remained within water window [38,40] and no adverse reaction was observed. This suggests the functionality of the hybrid probes for the used stimulation paradigms in clinical applications. 

### 4.4. Magnetic Resonance Imaging

The side by side comparison of the hybrid probe and a conventional DBS probe in MRI standard sequences showed imaging artefacts that exceeded the dimensions of the DBS probe by a factor between three and six and thus conceals the region of most interest. The hybrid probe, on the contrary, was displayed clearly and within its dimensions, without showing artefacts nor decreasing the imaging value in its vicinity. As has been reported earlier, these MR-imaging artefacts are eccentric of the electrode sites [28,56], which makes the localization of the electrodes imprecise and may affect the validation of the correct position negatively. The echo-planar imaging sequence commonly used for fMRI applications shows imaging artefacts twice the size of the other shown sequences, which is expected due to the higher sensitivity to image distortions caused by metal [31]. The here reported artefact size agrees with a report on artefacts extending approximately 1 cm from the DBS probe imaged in fMRI using a 3 T scanner [57]. Cunningham and co-workers claimed that the development of artifact-free electrodes would increase the applicability of fMRI [57]. Consequently, this highlights another advantage of the hybrid probe for studies aiming at gaining a better understanding of the brain where DBS is combined with fMRI. Further imaging of the hybrid probe should be performed in tissue to validate its visibility for localization purposes in vivo.

### 4.5. Limitations and Challenges

One may ask how these pilot results might serve in the development towards early clinical proof-of-concept/feasibility trials and what the next development steps should be. As the channel counts increases the task of interconnecting single channels to the implantable pulse generator (IPG) becomes more challenging. On the one hand, the aim is to miniaturize the dedicated space to the connection sides and on the other hand, the integrity of the connector (by means of mechanical and electrical aspects) should be kept intact. For the prototyped hybrid probes, the thin-film device was featured with 16 channels and the interconnection was realized using ZIF connectors. This approach can be followed up for acute clinical trials during surgical intervention. However, for chronic implantation, implementing more advanced interconnection technologies [58] is mandatory. These technologies require not only the compatibility with the hybrid probe but also they have to be in compliance with the changes in the interconnection technology to actual IPGs. Thin-film electrodes have proven their applicability and reliability in clinical trials [59,60] and so have the PDMS based electrodes in cardiac pacemakers [61] and all neuromodulation devices [62]. The hybrid probe is designed to benefit from both of these promising technologies. However, it must be mentioned that for a reliable manufacturing of the hybrid probe the interface between the PI and PDMS—as presented in this paper—is only suited for acute studies and has to be modified for enhanced adhesion and longevity in case of chronic applications. We have previously investigated the interface stability of the hybrid probe by applying a graded interface made out of silicon carbide and silicon dioxide to increase the adhesion strength of the polyimide to the underlying PDMS substrate [36,37]. The focus of this work was to investigate the influence of the spiral assembly in the hybrid design, introducing bending forces on the thin-film device on the performance of the incorporated glassy carbon. As a following step towards the clinical applications, besides addressing the needed technological improvements given in the discussion section, it is essential to evaluate the performance of the hybrid probe in the biological host environment in order to evaluate its longevity under the influence of the acute and chronic experiments.

## 5. Conclusions

The prototyped hybrid probe shows potential development pathways to increase channel count, reduce electrode size for higher spatial selectivity, and opens a window to integrate electrochemical neurotransmitter detection in addition to electrical recording and stimulation capabilities. Microsystems engineering offers the materials, technologies, and processes to manufacture functional structures. If combined it can be utilized in clinically implantable devices by using mandrels and hollow silicone rubber tubes. New diagnostic and treatment options can be further investigated and hence, structure and function of the brain can be further explored, not only on an anatomical but also on a multimodal/functional level. The way from technical designs studies and in vitro investigations to clinical trials and medical device approval is long and expensive. Applicability, functionality, and reliability have to be proven step by step in order to deliver novel tools and therapies using the latest technologies. Therefore, it is necessary to take advantage of the established materials and methods which are applicable to address the existing limitations and as a result, to bring innovative instruments as fast as possible into clinical applications. 

## Figures and Tables

**Table 1:** Comparison of the DBS lead parameters.

Parameter	Conventional DBS ^1^	DBS-Array Sapiens ^1^	Hybrid Probe
Diameter of the Lead	1.27 mm	1.27 mm	1.19 mm
Individual Contact Shape	ring	disc	disc
Individual Contact Size	1.50 mm	0.50 mm	50 μm
Circumferential Pitch	N.A.	90°	90°
Total Length of Array	7.5–10.5 mm	12.0 mm	10 mm
Total Number of Contacts	4	64	16
Biosensing Capability	no	no	yes

^1^ Data adapted from [11].
